# Investigations with Drugs and Pesticides Revealed New Species- and Substrate-Dependent Inhibition by Elacridar and Imazalil in *Human* and *Mouse* Organic Cation Transporter OCT2

**DOI:** 10.3390/ijms232415795

**Published:** 2022-12-13

**Authors:** Annett Kuehne, Saskia Floerl, Yohannes Hagos

**Affiliations:** PortaCellTec Biosciences GmbH, Science Park Va, Marie-Curie-Straße 8, 37079 Göttingen, Germany

**Keywords:** *SLC22*, OCT2, species differences, drugs, pesticides, elacridar and imazalil

## Abstract

Multiple drugs are used to treat various indications as well as pesticides that are ingested unintentionally and enter the bloodstream. The residence time or bioavailability of these substances in circulation depends on several mechanisms, such as drug–drug interaction (DDI), drug–pesticide interaction, metabolizing enzymes and the hepatic and renal transport systems, involved in the elimination of the compounds from the body. One of these transporters is the Organic Cation Transporter 2 (OCT2) member of the *solute carrier (SLC22)* transporter family. OCT2 is highly expressed in the proximal tubule epithelial cells in *human* and *mouse* kidney, where it mediates the uptake of endogenous organic cations as well as numerous drugs and xenobiotics, and contributes to the first step of renal clearance. In this study, we examined OCT2 on two subjects: First, the transferability of data from *mouse* to *human*, since *mice* are initially examined in the development of new drugs to assess the renal excretion of organic cations. Second, to what extent the choice of substrate affects the properties of an inhibitor. For this purpose, the functional properties of hOCT2 and mOct2 were validated under the same experimental conditions with the known substrates metformin and 1-Methyl-4-phenylpyridinium iodide (MPP). While hOCT2 and mOct2 showed very low affinities for metformin with K_m_ values of 3.9 mM and 3.5 mM, the affinity of hOCT2 and mOct2 for MPP (62 and 40 µM) was 64- and 89-fold higher, respectively. For our positive control inhibitor decynium22, we determined the following IC_50_ values for hOCT2 and mOct2: 2.2 and 2.6 µM for metformin uptake, and 16 and 6.9 µM for MPP uptake. A correlation analysis of the inhibitory effects of 13 drugs and 9 pesticides on hOCT2- and mOct2-mediated transport of metformin showed a correlation coefficient R^2^ of 0.88, indicating good interspecies correlation. Nevertheless, the bioenhancer elacridar and the fungicide imazalil showed species-dependent inhibitory potentials. Concentration-dependent inhibition of hOCT2- and mOct2-mediated metformin uptake by elacridar showed IC_50_ values of 20 µM and 1.9 µM and by imazalil 4.7 µM and 0.58 µM, respectively. In conclusion, although our data show comparable species-independent interactions for most compounds, there can be large species–specific differences in the interactions of individual compounds, which should be considered when extrapolating data from *mice* to *humans*. Furthermore, a comparison of the inhibitory potential of elacridar and imazalil on metformin uptake with that on MPP uptake reveals substrate-dependent differences in hOCT2 and mOct2 for both inhibitors. Therefore, it might be useful to test two different substrates in inhibition studies.

## 1. Introduction

The kidney as a multifunctional organ that has a particularly pivotal function in the elimination of urinary endogenous metabolites as well as exogenous substrates and their metabolites, and thereby in the clearance of plasma from numerous compounds. For the elimination of hydrophilic substances, the kidney has two different processes: First, an unselective glomerular filtration system and second, a highly selective, active transporter-mediated secretory pathway. Hydrophobic molecules also cross the membrane via the transporter-mediated pathway, which is highly dependent on the expression of specific transporters on the apical as well as on the basolateral side of renal tubular epithelial cells. The main process of secretion and absorption in the kidney takes place in the proximal tubular cells. Active secretion of positively or negatively charged organic compounds is mediated by transport proteins belonging to the *SLC22A* family. For the first step of the basolateral uptake of cationic compounds into proximal tubule epithelial cells, the Organic Cation Transporter 2 (OCT2, *SLC22A2*) is responsible. In contrast to OCT1, which is highly expressed in the liver, OCT2 is mainly expressed in the basolateral membrane of proximal tubules and at very low levels in lung ciliated epithelial cells, as demonstrated for *human* and rat by immunostaining [1,2,3,4]. OCT2 mRNA was detected at low levels in several tissues, but unlike OCT1, not in the intestine and liver [5]. The expression pattern of Oct2 in *mice* is comparable to that in *humans*, with the exception of the choroid plexus and adrenal gland [6]. The predicted membrane topology forms 12 transmembrane domains with the N- and C-terminal ends located in the intracellular space. Members of the *SLC22* family possess a relatively large extracellular loop between the first and second transmembrane domains. A model of the tertiary structure shows the inward conformation as described by Koepsell [7]. All OCTs have a similar number of amino acids (551 to 556). *Human* OCT2 protein shares 83%, 82%, 69%, 68%, 67% identity with *Mus musculus (m)* Oct2, *Rattus norvegicus* (r)Oct2, hOCT1, mOct1 and rOct1, respectively, while the amino acid sequence identity of hOCT2 to hOCT3 is less than 50%. *Human* OCT2 and its *murine* homologues as well as OCT1 have a similar substrate selectivity, which may be due to the high amino acid identity [5]. Unfortunately, little is known about the OCT2-specific species-dependent interaction with a variety of compounds. However, in 1940, Rennick and colleagues described the renal tubular secretion of organic cations in chicken, e.g., guanidine and N-methylnicotinamide (NMN) as well as renal secretion of tetraethyl ammonium (TEA) [8]. The renal secretion mediated by basolateral uptake and luminal excretion to the urine of positively charged compounds was observed in the kidneys of all examined mammals [9,10], snakes [11], fish [12,13] as well as invertebrates (crabs urinary bladder) [14], indicating an evolutionarily very highly conserved renal secretory system. The substrate selectivity between the species seems to be comparable, nevertheless a differential species dependent affinity was observed for MPP, TEA and NMN, as summarized by Burckhardt and Pritchard [15]. 

Since OCT2 plays a pivotal role in the pharmacokinetics of drugs and in drug–drug-interactions (DDI), it is recommended by the Food and Drug Administration (FDA) and European Medicines Agency (EMA) to be tested for new molecular entities (NME). Laboratory animals, particularly *mice*, are the first choice for initially assessing the clearance of new drugs, but it is often unclear how well pharmacokinetic and DDI data can be extrapolated from animal studies to *humans*. Consequently, an in vitro comparison of drug transporter interaction between transporter overexpressing *human* and *murine* cells will be very essential. Furthermore, to our knowledge, neither the interaction of hOCT2 nor mOct2 with pesticides has been systematically studied, although this is an interesting aspect for understanding effects of pesticides on renal secretion.

Pesticides are increasingly present in foodstuff, and the treatment of patients with multiple drugs is continuously increasing. Hence, the aim of this study was to compare the interaction of hOCT2 and mOct2 with 13 drugs as well as 9 pesticides, in order to identify substrate as well as species differences in the interaction with these compounds. The inhibitory potential of drugs to OCT2 mediated uptake of different substrates could highly vary, as demonstrated by Hacker et al. [16]. Thus, we examined and evaluated the inhibitory potential of drugs and pesticides, which are ingested with food or through occupational exposure, toward OCT2-facilitated uptake with two well-known OCT2 substrates metformin and MPP.

## 2. Results

### Functional Characterization of hOCT2 and mOct2

**Figure 1:**
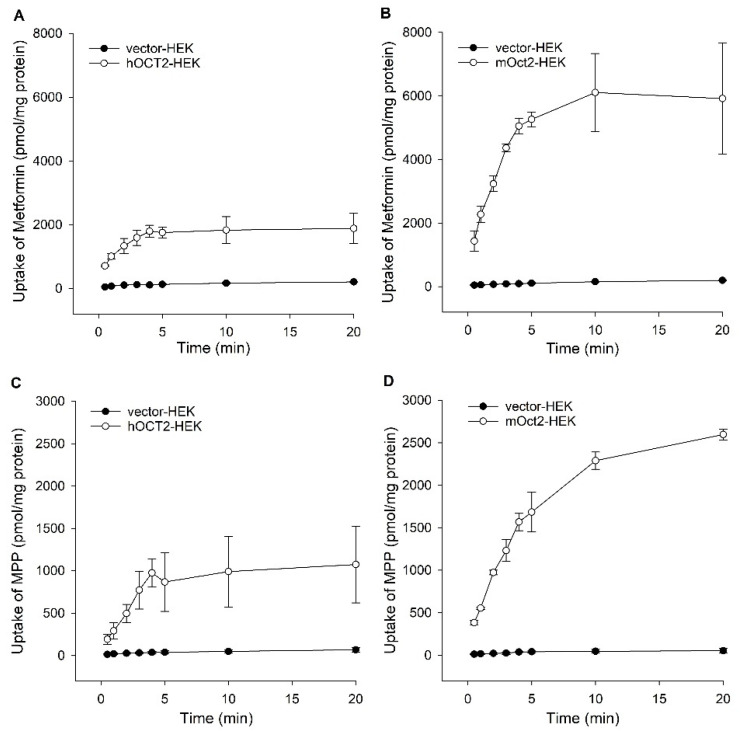
Time-dependent uptake of 100 µM metformin in (**A**) hOCT2-, (**B**) mOct2- 10 µM MPP in (**C**) hOCT2-, (**D**) mOct2-, and corresponding vector controls. Data are presented as the mean of two independent experiments carried out in triplicate ± average deviation.

To investigate hOCT2- and mOct2-facilitated uptake over time, transporter-HEK and control-HEK cells were incubated with metformin (100 µM) or MPP (10 µM) for eight time intervals ranging from 0.5 to 20 min. The hOCT2 and mOct2 mediated uptake of metformin as well as MPP was linear up to 4 min, after 5 min the uptake of both substrates was saturated in a comparable manner for hOCT2 and mOct2 (Figure 1A–D). The uptake ratios (uptake into transporter-HEK divided by control-HEK cells) were between 10 and 18-fold up to 4 min incubation.

**Figure 2:**
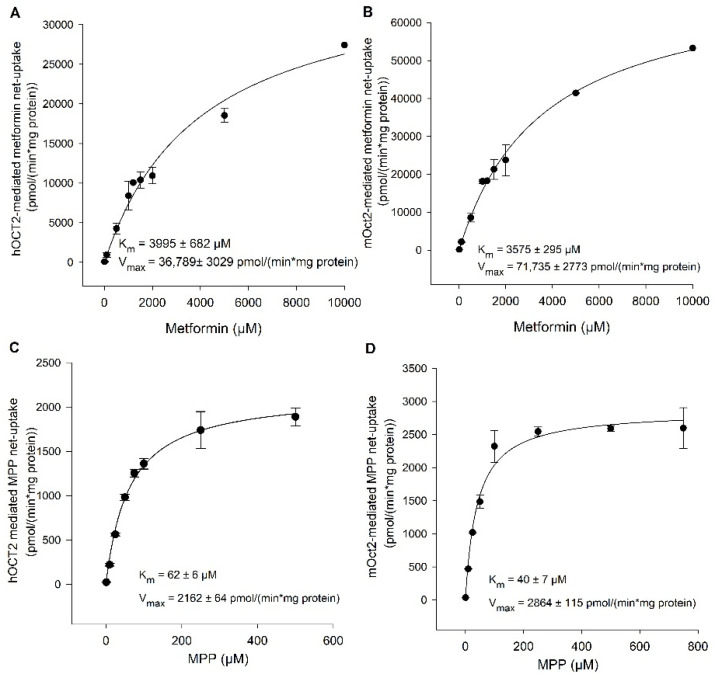
Kinetics of (**A**) hOCT2-, (**B**) mOct2-mediated ^14^C-metformin transport and (**C**) hOCT2-, (**D**) mOct2-mediated ^3^H-MPP transport. *Human* and *mouse* transfected HEK293 cells were incubated for 1 min at 37 °C in the presence of ^3^H-labeled (2 nM) and increasing concentrations of unlabeled MPP or 2 min in the presence of ^14^C-labeled (1 µM) and increasing concentrations of unlabeled metformin. Transporter specific net uptake was fit to the Michaelis–Menten equation to obtain the affinity constant K_m_ and maximum transport velocity V_max_ by non-linear regression analysis using Sigma Plot 13.0 software. Each data point represents the mean of two independent experiments carried out in triplicate ± average deviation.

To determine the affinity of *human* and *mouse* OCT2 for metformin and MPP, a concentration dependent uptake assay with eight substrate concentrations at one time point was carried out. The Michaelis–Menten kinetic analysis revealed lower affinity for metformin than for MPP observed in both OCT2-HEK cell lines (Figure 2). The K_m_ value for metformin was 3995 ± 682 µM for hOCT2 (Figure 2A) and 3575 ± 295 µM for mOct2 (Figure 2B), with V_max_ values of 36,789 ± 3029 and 71,735 ± 2773 pmol/(min*mg protein), respectively. Hence, the calculated K_m_ value for MPP with 62 ± 6 µM (Figure 2C) for hOCT2 and 40 ± 7 µM for mOct2 (Figure 2D) was 64-fold and 89-fold lower (higher affinity) compared to metformin. 

**Figure 3:**
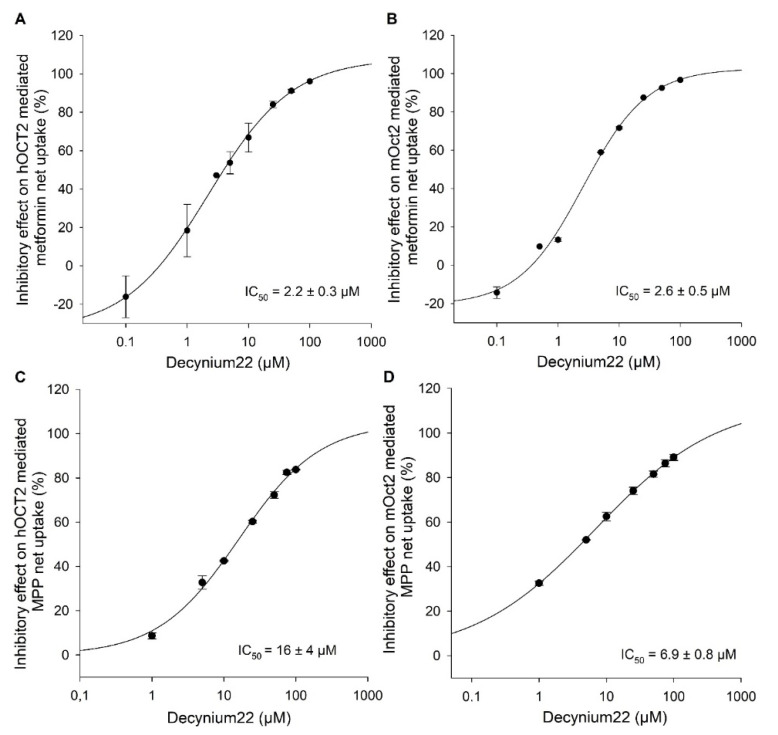
Concentration-dependent inhibitory effects of decynium22 on (**A**) hOCT2-, (**B**) mOct2-mediated ^14^C-metformin transport and (**C**) hOCT2-, (**D**) mOct2-mediated ^3^H-MPP transport in transfected HEK293 cells. Each data point represents the mean inhibitory effect (%) calculated from net-uptake of two independent experiments carried out in triplicate ± average deviation. IC_50_ values were calculated by sigmoidal 4Hill equation using Sigma Plot version 13.0 software.

For further characterization of the two cation transporter systems, the inhibitory potential of decynium22, a well-known inhibitor of OCTs, was evaluated by inhibition of hOCT2- and mOct2-mediated metformin as well as MPP uptake. As depicted in Figure 3, the transporter mediated metformin uptake was highly inhibited by decynium22, with calculated IC_50_ values of 2.2 ± 0.3 µM (Figure 3A) and 2.6 ± 0.5 µM (Figure 3B), whereas the MPP uptake was less inhibited by decynium22, with calculated IC_50_ values of 16 ± 4 µM for hOCT2 (Figure 3C) and 6.9 ± 0.8 µM for mOct2 (Figure 3D), respectively.

The basic functional transport data time dependency, K_m_ and V_max_ values for the two substrates showed no relevant species-dependent differences in hOCT2-HEK and mOct2-HEK cells. However, for decynium22 used as positive control inhibitor, the IC_50_ value determined with MPP as substrate showed a slightly higher value (2-fold) for *human* than for *mouse* OCT2. 

After initial characterization of hOCT2 and mOct2-HEK cells with their respective control-HEK cells, we assessed the interaction of thirteen cationic drugs and nine pesticides with both transporters. For this purpose, we incubated the cells with the substrate metformin together with 10 or 100 µM of the drugs or pesticides. The accumulation of radio-labeled metformin in OCT2-HEK and control HEK cells was determined and from this the percentage inhibitory effects of the individual compounds were calculated. As summarized in Table 1, cyclosporin A, cimetidine, amiodarone, reserpine, quinidine, ranitidine, ritonavir, procainamide, and zosuquidar did not show a relevant inhibition of metformin uptake by *human* and *mouse* OCT2. However, some compounds caused a stimulatory effect (calculated as negative inhibitory effects) on the metformin transport. The highest inhibitory effect (over 50%) on hOCT2- or mOct2- mediated metformin uptake at 100 µM was observed for decynium22 (control inhibitor), followed by ketoconazole, clonidine, elacridar, and verapamil in that order. The inhibition of hOCT2- and mOct2-mediated uptake by decynium22, ketoconazole, clonidine, and verapamil was comparable and did not show any species dependent differences. Interestingly, for elacridar (GF120918), we determined a two-fold higher inhibitory effect at the lower concentration of 10 µM in mOct2-HEK than in hOCT2-HEK cells (Table 1).

The pesticides paraquat, glyphosate, atrazine, imidacloprid, amitraz, prochloraz and azoxystrobin showed very low inhibitory effects at 100 µM on metformin accumulation in both OCT2 expressing cells (Table 2). Even so, some pesticides induced a stimulatory effect on the metformin uptake (Table 2), particularly amitraz on hOCT2. However, propamocarb and imazalil demonstrated the highest inhibition of hOCT2- or mOct2-mediated metformin uptake. Propamocarb at 100 µM showed inhibitory effects of 55% in hOCT2 and of 48% in mOct2, respectively. At 10 µM, the influence of propamocarb was negligible. In contrast, imazalil at the lower concentration of 10 µM showed 52 and 83% inhibitory effects on hOCT2- and mOct2-mediated metformin uptake. At the highest concentration, the hOCT2- and mOct2-mediated metformin uptake was comparably abolished by imazalil as described for the control inhibitor decynium22 (Table 2).

**Figure 4:**
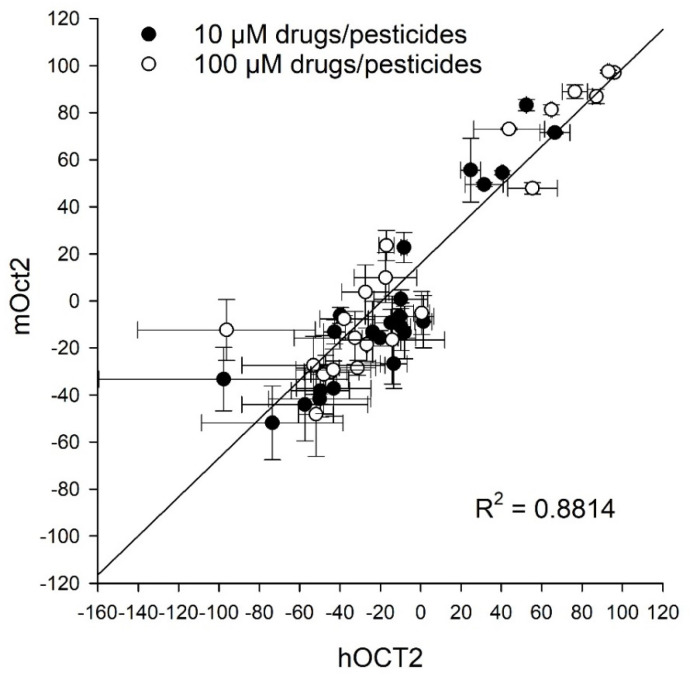
Correlation analysis between inhibitory effects of hOCT2- and mOct2-mediated transport of metformin. A correlation coefficient (R^2^) of 0.88 indicates a good correlation between species. Data points represent mean and average deviation of two independent experiments. Mean values in detail are presented in Table 1 and Table 2.

The results of the inhibitory effects with the drug metformin were further evaluated by correlation analysis. As depicted in Figure 4, the calculated correlation coefficient R^2^ of 0.88 indicates a very high functional similarity of hOCT2 and mOct2 in interaction with the most examined drugs and pesticides. Nevertheless, a slight species dependent difference of elacridar and imazalil was observed. 

**Figure 5:**
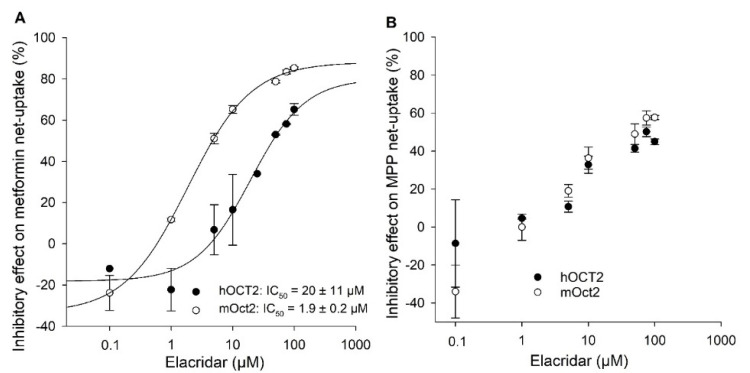
Concentration-dependent inhibitory effects of elacridar on hOCT2- and mOct2-mediated metformin (**A**) and MPP (**B**) transport in transfected HEK293 cells. Each data point represents the mean inhibitory effect (%) calculated from net-uptake of two independent experiments carried out in triplicate ± average deviation. IC50 values were calculated by sigmoidal 4Hill analysis using Sigma Plot version 13.0 software.

Therefore, an IC_50_ value for both compounds was determined. Concentration-dependent inhibition of hOCT2- and mOct2-mediated metformin transport by elacridar resulted in IC_50_ values of 20 ± 11 µM and 1.9 ± 0.2 µM, respectively (Figure 5A). 

**Figure 6:**
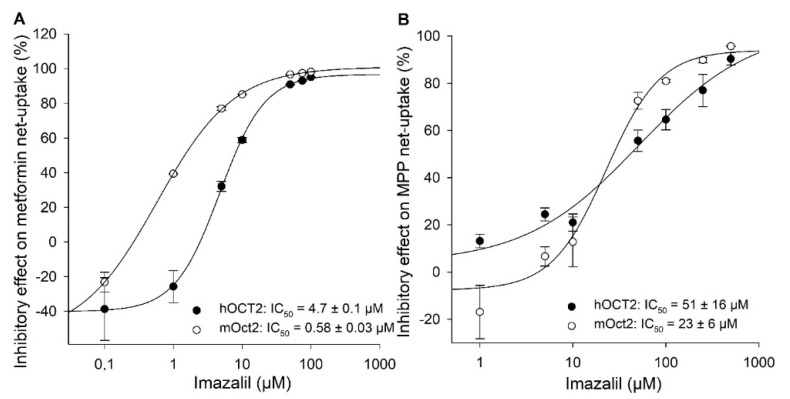
Concentration-dependent inhibitory effects of imazalil on hOCT2- and mOct2-mediated metformin (**A**) and MPP (**B**) and transport in transfected HEK293 cells. Each data point represents the mean inhibitory effect (%) calculated from net-uptake of two independent experiments carried out in triplicate ± average deviation (AD). IC50 values were calculated by sigmoidal 4Hill equation using Sigma Plot version 13.0 software.

The IC_50_ value for imazalil was determined to be 4.7 ± 0.1 µM for hOCT2 and 0.58 ± 0.03 µM for mOct2 (Figure 6A). Hence, with metformin as substrate, both elacridar and imazalil showed higher inhibitory potency (11-fold and 8-fold) to *mouse* than to *human* OCT2. 

To investigate whether the inhibitory effects (IC_50_ values) of elacridar and imazalil are substrate-dependent, the uptake of MPP as a second substrate with a higher affinity for both OCTs was investigated with increasing concentrations of elacridar or imazalil. (Figure 5B and Figure 6B). Since the highest soluble concentration of elacridar in assay buffer was 100 µM (determined visually), MPP uptake could be inhibited by a maximum of 60% and, therefore, no IC_50_ value could be determined for either OCT2 transporter (Figure 5B). The calculated IC_50_ value of 51 ± 16 µM for imazalil for hOCT2-mediated MPP uptake was 2-fold higher compared to mOct2 (23 ± 6 µM; Figure 6B), a much smaller difference than the 8-fold difference observed with metformin as substrate.

In summary, for the control inhibitor decynium22 as well as for elacridar and imazalil, the IC_50_ values were lower with the drug metformin as substrate compared to the second substrate MPP. Furthermore, the IC_50_ values were lower in *murine* than in *human* OCT2 for elacridar and imazalil. Thus, we showed species-dependent differences for 2 and substrate-dependent differences for 3 out of 23 investigated compounds.

## 3. Discussion

The renal clearance, beside the liver, is the main system for eliminating endogenous metabolites as well as exogenous compounds ingested through food and drugs. Especially hydrophilic compounds are partially eliminated in the kidney by active transporter-mediated transcellular secretion. The proximal tubule possesses a number of different and specific secondary or tertiary active transporter proteins. After the functional characterization of the OCTs over the last three decades, the physiological, pathophysiological and pharmacological importance and the clinical implications have been appreciated and recognized by the regulatory agencies (FDA and EMA), for OCT2 in particular. In drug development, the initial preclinical tests are conducted with laboratory animals. Therefore, *mice* are an indispensable part of the new molecular entities (NME) development to identify the renal secretory systems for anionic or cationic organic compounds. In this renal secretory system, mOct2 plays a very important role. The main objectives of this study were, first, to examine OCT2 in terms of the transferability of data from *mice* to *humans* and second, to investigate to what extent the choice of substrate influences the properties of an inhibitor. 

The affinity of hOCT2 and mOct2 for metformin was 64- and 89-fold lower than for MPP. This huge difference in affinity of the OCTs for metformin and MPP could be explained by the structural properties of the OCTs with a high-affinity and a low-affinity binding site [17,18].

Several working groups [19,20,21,22] reported affinity values (Km, Kt values) for hOCT2 that were 3- to 6-times lower than the Km values determined in this study. A direct comparison of the experimental conditions has shown a difference in the buffers systems. While our study was performed in HBSS buffer with 5 mmol/L bicarbonate, the other studies were performed in buffers without bicarbonate (e.g., Waymouth buffer). Goralski et al. 2002 described the effect of bicarbonate on the rOct1- and rOct2-mediated interaction with radiolabeled substrates [22]. We could not observe this effect with the substrate metformin, since the calculated Km value (3995 µM) in this study is comparable to that of Zolk et al. (3356 µM) [22].

Regarding mOct2, we demonstrated, for the first time, the affinity for metformin and MPP in overexpressing HEK293 cells. As to our knowledge, no functional or kinetic data for mOct2 on metformin or MPP uptake in overexpressing cells were previously available In vivo experiments with wild type and knockout *mice* emphasized the crucial role of mOct2 in renal secretion of numerous compounds, e.g., the group of Ciarimboli et al. showed significantly reduced creatinine, metformin and TEA clearance with Oct1/Oct2 knockout *mice* [23,24]. However, in the single Oct2 knockout, the TEA clearance was not different from that in wild type *mice*. Surprisingly, the MPP clearance in Oct1/Oct2 knockout compared to wild type *mice* was not affected [25]. The systemic relevance of mOct2 for corticosterone levels in the brain and plasma and a contribution of genetic polymorphism of OCT2 were summarized in the review of Kölz et al. [26]. 

Decynium22 is a well-known inhibitor of OCTs as well as the plasma membrane monoamine transporter (PEMAT), which has been discussed as a potential drug for the treatment of schizophrenia and depression since it is known to increase the extracellular serotonin level in *mice* brain [27]. The inhibitory potential of decynium22 on metformin uptake of both transporters was 7.2- and 2.7-fold higher compared to MPP uptake. The lower IC_50_ values for decynium22 by the OCT2 facilitated uptake of metformin could be explained by the very low affinity of OCT2 for metformin. While several other groups reported IC_50_ values for decynium22 interaction with hOCT2, comparable data for decynium22 in mOct2-HEK cells are not available. Here, we report, for the first time, the IC_50_ values for decynium22 by inhibition of metformin as well as MPP uptake in mOct2. For the MPP uptake in hOCT2-HEK293 cells, Hayer-Zillgen et al. 2002 reported an IC_50_ value of 1.3 µM for decynium22 [28]. A lower IC_50_ value (0.1 µM) of decynium22 was determined for hOCT2-mediated TEA uptake [29]. These data indicate that IC_50_ values depend on the substrate used for the uptake assay.

In summary, the basic functional transport data time dependency, K_m_ and V_max_ values showed no relevant species-dependent differences in hOCT2-HEK and mOct2-HEK cells. The IC_50_ values for decynium22 using MPP as substrate in hOCT2 and mOct2 revealed a slight (2.3-fold) species dependent difference, while the decynium22 IC_50_ value for metformin uptake was identical for the two species.

The interaction study with various drugs brought the following findings regarding species and substrate dependency: The drugs cyclosporin A, reserpine, zosuquidar, amiodarone, ritonavir, quinidine, procainamide, and ranitidine showed even at 100 µM no inhibitory potential but rather a stimulatory effect, as observed also for *human*, *rat* and *mouse* OCT1 [30]. Literature data also showed that transcellular transport (basolateral to apical) of metformin was not inhibited by cyclosporine [31]. Reserpine has been described as a specific inhibitor of the vesicular monoamine transporter (VMAT-2) [32], but it does not inhibit OCTs. Amiodarone has a very high inhibitory potential (K_i_ = 5.7 µM) for OCTN2, another member of the *SLC22A* family. For ritonavir, Wittwer et al. reported the inhibitory effect on hOCT1-, hOCT2- (IC_50_ = 24.8 µM), and MATEs- (IC_50_ = 4.4 µM) mediated ASP^+^ uptake [33]. In this case, a possible explanation for the discrepancy with our data is the use of a different substrate. Quinidine showed at our hand at 10 µM no inhibitory effect on the metformin uptake by both hOCT2 and mOct2. In contrast, several groups demonstrated an inhibition of hOCT2- mediated uptake of MPP by quinidine with IC_50_ values at approximately 90 µM, determined in CHO cells [19,34]. The experimental difference could be the reason for the contradictory results. Inhibition of TEA (IC_50_ = 50 µM) and YM155 (IC_50_ = 92 µM) uptake by procainamid in OCT2-overexpressing oocytes as well as HEK293 cells was reported, respectively [29,35]. A stimulation of metformin uptake by OCTs was observed with ranitidine in our studies. Interestingly, Tahara et al. reported that ranitidine is a substrate of hOCT2 (K_m_ = 65.2 µM) and furthermore, ranitidine showed inhibition of cimetidine uptake by hOCT2 but not by rOct2 [36]. In the same study, ranitidine inhibited species independently of the hOCT2- and rOct2-mediated famotidine uptake. 

Elacridar, ketoconazole, clonidine, and decynium22 are potent inhibitors of OCT2. Elacridar is a very well-known inhibitor of MDR1 and BCRP [37,38], but the interaction with the *SLC22A* transporter family has not been reported so far. We observed a species dependent inhibitory potential of elacridar on hOCT2- and mOct2-mediated metformin uptake. The calculated IC_50_ values showed a 10-fold higher affinity for mOct2 in comparison to hOCT2 (1.9 µM versus 20 µM). In contrast, the inhibitory potential of elacridar on the MPP uptake by OCT2s was very low and a calculation of an IC_50_ value was not possible. However, this shows all the more the large differences that can occur when using different substrates. 

Ketoconazole is a multi-specific inhibitor of numerous transporters (MDR1, MRP2, OATs, OATPs, MATEs and OCTs), as reported by several groups and particularly by Vermeer et al. who determined an IC_50_ value of 0.89 µM for hOCT2 [39]. We determined no IC_50_ values for ketoconazole but the inhibitory effects with 10 µM were 31% for hOCT2, 49% for mOct2, and 87% for both transporters with 100 µM. Clonidine is apparently a very potent inhibitor of hOCT2 and mOct2. The reported IC_50_ value for hOCT2-mediated MPP or metformin uptake was 6 µM and 0.68 µM, respectively [19]. We observed a reduction of metformin uptake by 60% in hOCT2 as well as by 46% in mOct2 with 10 µM clonidine, which is in accordance with the reported data for hOCT2 and clonidine. 

*Humans* as well as animals are unfortunately highly exposed to pesticides through food intake and environmental pollution. Particularly occupationally exposed people are at a high risk of accumulating large amounts of pesticides in the body, so the Admissible Daily Intake (ADI) as well as the Maximum Residue Level (MRL) defined by regulatory agencies are helpful parameters to protect people from harmful accumulation of pesticides. Other aspects to consider are the impact of pesticides on absorption, distribution, metabolism and elimination (ADME) and thus the pharmacokinetics of drugs as well as endogenous compounds or metabolites. Transport proteins are the bottleneck in the absorption and elimination of compounds in the body. Interactions of pesticides with transporters can affect drug ADME processes. Therefore, the understanding of transporter proteins in handling with the pesticides is of immense importance. Unfortunately, very little information is available on the interaction of pesticides with SLC-Transporters. One aspect of this study was to better understand the role of pesticides in specific transporter-mediated absorption and systematic elimination through secretory systems. Previously, we reported the interaction of several pesticides with OCT1, NTCP, and OAT1 [30,40,41]. Particularly, Floerl et al. described the renal secretion of several pesticides as well as the MRL levels of some pesticides and their impact for consumers or occupationally exposed people or suicidal intentional ingestions [30]. In this study, we examined the interaction of 9 pesticides with *human* and *mouse* OCT2, which enabled us to identify species dependent differences. Nine structurally diverse but mainly positively charged (at pH 7.4) pesticides were selected and their inhibitory potential on hOCT2- or mOct2-facilitated metformin uptake was evaluated in stably transfected HEK293 cells. In our study, the metformin uptake by OCTs was not inhibited by paraquat, although for *human* OCT2, a concentration dependent uptake of paraquat (K_m_ value of 114 µM) was reported [42]. Numerous of the pesticides are renally excreted and detectable in the urine, as previously described. However, the active secretory mechanism beside the glomerular filtration of the most pesticides, such as glyphosate, imazalil, azoxystrobin, atrazine, amitraz as well as imidacloprid are not clear. Inhibition of hOCT2- and mOct2-mediated uptake was only observed for imazalil and propamocarb. Comparable inhibitory potential of imazalil and propamocarb on hOCT1-, mOct1-, and rOct1-mediated MPP uptake was previously reported [30]. Additionally, MATE1-mediated inhibition of metformin uptake by imazalil was demonstrated by Floerl et al. 2020 [30]. Propamocarb inhibited the hOCT1 as well as hOCT2 activity and stimulated the MATE2K [43]. Therefore, basolateral uptake by OCT1 and OCT2 and the luminal secretion by MATE1 presumably contribute to renal imazalil and propamocarb clearance through transcellular active secretion.

In conclusion, *murine* transporter systems are reasonable models for initial characterization of NME interaction with specific membrane transporters and provide a good basis for extrapolating data to *human* orthologous transporter systems. However, particular compounds demonstrate species dependent interactions, as shown for elacridar and imazalil. Additionally, a substrate dependent inhibition of hOCT2 and mOct2 by elacridar, imazalil, and partially for decynium22 was observed. Consequently, species and substrate dependent differences should always be considered when extrapolating data for NME from *mouse* to *human*. 

## 4. Materials and Methods 

### 4.1. Material and Cell Lines

^14^C-metformin (1,1-Dimethylbiguanide hydrochloride) and ^3^H-MPP (1-Methyl-4-phenylpyridinium iodide) were purchased from American Radiolabeled Chemicals, St. Louis, MO, USA. All non labelled chemicals were obtained from Sigma-Aldrich, Darmstadt, Germany. 

With cDNA of hOCT2 (GeneBank: accession number: NM_003058.3) or mOct2 (NM_013667.2), stably overexpressing HEK293 cells were used for the experiments and empty vector-transfected HEK cells were used as control-HEK cells. 

All HEK-293 cell lines (hOCT2-, mOct2 and the vector-HEK-cells) were routinely tested for mycoplasma using Mycoplasma Detection Kit (Venor^®^GeM, Minerva biolabs, Berlin, Germany) for conventional PCR and used if free from mycoplasma.

### 4.2. Transporter Mediated Uptake of Radiolabeled Substrates 

The uptake assays were performed as previously described [41] in 24-well plates. Therefore, culture medium was aspirated, and each well was rinsed three times with 0.5 mL incubation buffer (HBSS supplemented with 20 mM HEPES, pH 7.4) and then incubated in 200 µL dosing solution containing ^3^H-MPP or ^14^C-metformin and the test compound. After incubation, the uptake was stopped by aspirating the reaction mixture and washing the cells three times with 0.4 mL ice-cold PBS buffer. Cells were then solubilized with 0.6 mL of 1 N NaOH, overnight. The whole content of each well (0.6 mL) was transferred to a scintillation vial (Perkin Elmer). The radio-labelled amount was determined by liquid scintillation counting.

For time dependent uptake of metformin as well as MPP, the hOCT2-HEK and mOct2-HEK as well as control-HEK cells were incubated with 10 µM MPP (2 nM [^3^H] MPP) or 100 µM metformin (1 µM [^14^C] metformin) at eight time intervals (0.5 to 20 min).

To determine the affinity (K_m_ value) for metformin and MPP as known substrates of organic cation transporters, saturation experiments at an initial linear period were performed as determined in time dependency experiments for 2 min for all metformin experiments and for 1 min for all MPP experiments. *Human* and *mouse* OCT2-HEK and control-HEK cells were incubated with 1 µM [^14^C] metformin or 2 nM [^3^H] MPP and increasing concentrations of non-labeled metformin (10–10,000 µM) or MPP (1–750 µM). All experiments were conducted on two separate days in triplicate. 

The cellular protein amounts were analyzed in parallel to the transport experiments by the Bradford method [44] as previously described [41].

### 4.3. Inhibition Experiments 

Inhibition experiments for IC_50_ determination of the known inhibitor of organic cation transporter, decynium22, were performed at a 10-fold lower metformin concentration of 350 µM at the respective calculated K_m_ values of MPP. The metformin or MPP uptake was cis-inhibited by 0.1–100 µM Decynium22. For IC_50_ determination of elacridar and imazalil 1 µM [^14^C] metformin uptake and 2 nM [^3^H] MPP uptake was inhibited by 0.1–100 µM elacridar and 0.1–500 µM imazalil. All inhibition experiments were conducted on at least two separate days in triplicate. 

For screening experiments, cis-inhibition was carried out in duplicate by measuring the uptake of the labeled probe substrate in the absence and presence of 10 µM or 100 µM of the respective pesticide or drug. Transporter- and vector transfected HEK293 cells were incubated for 2 min with 1 µM [^14^C] metformin. Inhibitory effects in percent were calculated from net-uptake.

### 4.4. Data Analysis

For the Km calculation of metformin or MPP the transporter mediated uptake (pmol/mg protein/min) was plotted against substrate concentrations. The Km and Vmax values were obtained using SigmaPlot version 13 by fitting the Michaelis–Menten equation V = V_max_*[S]/(K_m_ + [S]), where V refers to the rate of substrate transport, V_max_ refers to the maximum rate of substrate transport, [S] refers to the concentration of substrate, and K_m_ is defined as the concentration of substrate at the half-maximal transport rate. The inhibitory effect I (%) was calculated according to the formula: I(%) = 100 − (V_with inhibitor*_100/V_w/o inhibitor_) and for the IC_50_ calculation of the inhibitor, the inhibitory effect I (%) was plotted against inhibitor concentrations and fitted using four parameter Hill equation using SigmaPlot 13.

## Figures and Tables

**Table 1:** Inhibitory effects (mean ± average deviation (AD)) of various drugs on hOCT2- and mOct2-mediated metformin-uptake. * Decynium22 is a control inhibitor for OCTs.

Drugs/Pos. Control Inhibitor *	Type of Drug	Charge at pH 7.4	Inhibitory Effects (%)							
			hOCT2				mOct2			
			10 µM		100 µM		10 µM		100 µM	
			Mean	AD	Mean	AD	Mean	AD	Mean	AD
Zosuquidar	antineoplastic drug	37% uncharged 63% cation	−10	5	−47	11	−11	10	−30	7
Procainamid	class I antiarrhythmic agent	100% cation	−20	9	−44	14	−16	3	−30	0
Ritonavir	antiretroviral HIV	100% cation	−10	13	−38	12	1	4	−8	1
Ranitidine	H2 histamine receptor antagonist	100% cation	−43	18	−33	30	−37	12	−16	11
Quinidine	class I antiarrhythmic agent	100% cation	−50	14	−31	9	−38	11	−28	3
Amiodarone	class III antiarrhythmic agent	100% cation	−10	17	−27	12	−7	0	4	12
Reserpine	hypertension	70% uncharged 30% cation	−13	7	−27	1	−27	11	−19	7
Cimetidine	H2 histamine receptor antagonist	75% uncharged 25% cation	−50	25	−17	4	−42	2	24	6
CyclosporinA	immunsuppressant	100% cation	−8	1	0	4	−13	11	−5	9
Verapamil	class IV antiarrhythmic agent	100% cation	−8	2	44	18	23	6	73	0
Elacridar	bioenhancer targeting drug resistance in tumors	100% cation	25	5	65	0	56	14	81	2
Clonidine	hypertension	100% cation	40	0	76	6	54	1	89	3
Ketoconazole	antifungal	82% uncharged 18% cation	31	9	87	2	49	1	87	3
Decynium22 *	inhibitor for cation transporter	100% cation	67	8	96	0	72	1	97	0

**Table 2:** Inhibitory effects (mean ± average deviation (AD)) of various pesticides on hOCT2- and mOct2-mediated metformin-uptake.

Pesticides	Type of Pesticide	Charge at pH 7.4	Inhibitory Effects (%)							
			hOCT2				mOct2			
			10 µM		100 µM		10 µM		100 µM	
			Mean	AD	Mean	AD	Mean	AD	Mean	AD
Amitraz	insecticide	100% cation	−98	62	−96	44	−33	13	−12	13
Atrazin	herbicide	100% uncharged	−57	31	−53	35	−44	15	−27	12
Paraquat	herbicide	100% cation	−15	8	−52	9	−9	6	−48	18
Glyphosat	herbicide	73% anion 27% ± charge	−24	1	−48	6	−13	13	−31	17
Imidacloprid	insecticide	100% ± charge	−74	35	−43	18	−52	16	−29	9
Prochloraz	fungicide	100% cation	−43	5	−17	15	−13	5	10	11
Azoxystrobin	fungicide	100% uncharged	−40	2	−14	26	−6	3	−17	19
Propamocarb	fungicide	100% uncharged	1	4	55	12	−9	11	48	2
Imazalil	fungicide	81% uncharged 19% cation	52	0	93	0	83	2	97	1
